# Cases and Observations on the Use of Strychnia in Intermittent Fevers

**Published:** 1848-02

**Authors:** J. E. McGirr

**Affiliations:** Chicago


ILLINOIS AND INDIANA

MEDICAL AND SURGICAL JOURNAL.

Vol. II. NEW SERIES.—FEBRUARY, 1848. No. 6.

### PART I.—ORIGINAL COMMUNICATIONS.

### ARTICLE I.

*Cases and Observations on the use of Strychnia, in Intermittent Fevers.* By J. E. McGirr, A. M., M. D., Chicago.

There is perhaps no practitioner, who has resided for any length of time in those districts where Intermittent Fevers prevail, but has met with cases of agues, more commonly old and sometimes even recent agues, in which his best directed efforts were powerless to restore deranged secretion or perverted nutrition. He has been compelled to see the sallow and emaciated being, perhaps a near and dear friend, wearing daily away, until the hollow cough and the sunken and lustreless eye have shown too plainly that the days of the victim were few for this world. Having met with such in my practice in the Juniata district, of Pennsylvania, it was with no small degree of satisfaction that I first learned from Professor Brainard, the advantages he has derived from the use of Strychnia in cases of this nature, in the Chicago Hospital, under his direction. I witnessed the salutary effects of its use and resolved to try it if an opportunity offered. The following are cases in which I have tried it and they will show for themselves. I would merely premise that the crystals of Strychnia were used.

Case 1.—Mrs. F., æt. 28, labourer's wife, whose health previously to her attack of ague had been good, and whose habits were regular, placed herself under my care on the 15th of June last. She states that she has had the ague for several months, thinks since October of last year, at intervals, and she used Sappington's pills, which mostly arrested it, for a few days, but they afforded her no permanent relief. She has now the ague every second day; the shake occurring about 10 o'clock, A. M. Her tongue is coated white; skin harsh and dry; head-ache and general lassitude; bowels irregular; great thirst and occasional vomiting; spleen enlarged; breathing oppressed; much emaciated and without any appetite. On percussing, the chest sounds clear; auscultating, the breathing is found distinct over the whole chest. She is nursing a child four months old.

I ordered her quinine, ten grains, with one-sixth of a grain of sulphate of morphia, to be taken, night and morning, for four days.

16th. She feels much better. Her shake to-day was much lighter than usual, but the quinine lies heavy upon the stomach and was to-day rejected. The spleen is much reduced in size. I may here state that M. M. Valleix and Gouraud, think that quinine has no effect upon the volumn of the spleen in ague—London Lancet, vol. 6, p. 397—while M. Piorry contends that it has. Whether it was the remedy that reduced the size of the spleen, in this case, I will not say; but that it was reduced and that no other remedy was exhibited but that ordered, I unhesitatingly assert, and my opinion is with those who believe in its efficacy, in this respect. As the stomach did not bear the quinine well, I for the first time ordered the Strychnia, in the manner recommended by Professor Brainard, namely: one-eighth of a grain, three times, daily, a dose to be given after each meal. He says, (Journal, vol. 2, p. 118,) "that when it is mixed directly with the contents of the stomach, it is less likely to produce unpleasant effects."

20th. Feels better than she has done since her first attack. The shake has left her, as also, the headache; tongue clean; bowels regular; able to sit up again, and appetite better. Strychnia to be continued until she takes 2 grains more.

I saw her on the 20th of October up to which time she had continued well and without a relapse.

Case 2.—J. C., æt. 34, a short, thick set, labouring man of regular habits, and whose health previous to his attack of ague had been excellent, applied to me on the 30th of August. He says that he has had the ague since May, and that he has been able to check it with quinine, for a week or ten days, but that it would always return again. His present condition is, impaired digestion; bowels lax; countenance blanched; tongue coated and white; pain over the eyes; skin dry and harsh; urine scanty, deep red, and scalding; pulse irregular and quick; shake every day in the forenoon. Percussion and auscultation, found nothing irregular to note in the chest.

As he complained that he felt a chill coming on, I ordered him twenty grains of quinine, and to return on the first of September.

Sept. 1st. One-eighth of a grain of Strychnia ordered three times each day.

5th. Shake arrested; head much better; bowels more regular; urine improved in quantity and appearance; pulse more regular; appetite not much better. I directed him to continue Strychnia until he had taken two grains more.

16th. He came back to-day with a return of his disease; he had eaten a portion of water-mellon and was immediately attacked with the ague. I gave him fifteen grains of quinine and one-sixth of a grain of morphia to be taken to-night, and the same quantity for to-morrow morning.

17th. Had no shake to day; feels better; but his appetite is very poor; I ordered him the Strychnia as before.

19th. Feels much better; appetite a little better; says the medicine jerks his arms a little, and that it vomited him a little the first time he took it. I directed its continuance.

Oct. 7th. Disease returned in consequence of getting wet. I ordered him again fifteen grains of quinine and one sixth grain of morphia.

8th. No shake to-day. I ordered him two grains of Strychnia to be divided into sixteen papers, one to be taken after each meal until eight are taken, then two each day till the remaining eight are taken.

15th. Better than he has been since May last; says he feels this time as if the ague had left, but that he felt before as if it would return again. Appetite good, and color improving. Three doses of the remedy cause him to have slight tetanic spasms, two do not at all effect him. I ordered him two grains more Strychnia, to be taken as by the last direction, but with an interval of four days between each grain.

I saw him a few days ago, though he has been labouring in all kinds of weather, and sometimes for hours in the water, he has not the slightest evidence of a relapse.

Case 3. H. W. æt. 28, a carpenter, of regular habits, placed himself under my care on the 15th of September. He says that he has had remittent fever, by which he was confined to bed for six weeks, and since he has got up he is gaining no strength, but is rather losing the little that he had. For a while he had a voracious appetite, but eating did not appear to benefit him. He has now no appetite, and on the least exertion, even walking over the floor of his room, he feels as if he would never recover from the exhaustion. Bowels are loose; abdomen tympanitic; occasional watery stools, tinged with blood; countenance sallow; is much emaciated; breathing quick, pulse 120 and small; tongue deeply coated, with red tip and edges. Percussion gives a clear sound over the chest; auscultation, the *bruit de suffle* in the carotids. He shakes every second day, about 10 o'clock, A. M., and the fever continues on him until about 4 o'clock, P. M. I ordered him to take one-twelfth of a grain of Strychnia after each meal.

17th. The only perceptible change is, that the fever lasted a shorter time, to-day, than usual. Strychnia to be continued.

19th. Feels a little better; diarrhœa not so troublesome; shake not quite so severe. Continue Strychnia.

22d. Is much better; escaped one day without the shake, but had a chill that was not very marked, though some fever followed; diarrhœa has nearly ceased; appetite returning. Increase the dose of Strychnia to one-eighth of a grain.

30th. Had no chill or fever since the twenty-second; diarrhœa disappeared entirely; appetite pretty good. He was able to nail some boards on his garden fence, to-day, without much exhaustion. I directed him two grains more of Strychnia, one half to be taken in four days, and the remaining half in like manner, after an interval of four days more.

Oct. 18.. Feels as well as ever he did.

Case 4.—Mrs. T's. servant girl, æt. 16, sent to-day, Oct. 18, for some medicine for dysentery. I sent her of comp. syrup of Hœmatoxylon and Rheubarb, two ounces, of Hydrochlorate of morphia, two grains, mixed, with directions to take one teaspoonful three times a day, or oftener, if the pain and tenesmus were severe.

26th. She came to tell me that the dysentery had not been checked, although she had taken all the medicine. The mitigation of the pain, was all the benefit she experienced. She has chills and fevers every day; appetite gone entirely; has a great deal of pain in the abdomen, and severe headache every evening; so much so, as nearly to blind her. I ordered her two grains of Strychnia to be divided into sixteen papers; one, to be taken after each meal, until eight are taken; then, two each day, until the remaining eight are taken.

Nov. 18th. Came to pay her bill, and says that the dysentery, chills and fever, stopped on the third day after she began to take the powders. She had no relapse.

Case 5. O. B., emigrant, æt. 40, enjoyed excellent health until he arrived in Chicago. He put himself under my care on the 17th of November. He had the fever for the last six weeks. His disease began as remittent fever, and changed to intermittent. Has a shake every second day, which begins about two o'clock, P. M., when he is obliged to go to bed, and does not get warm until next morning.

Percussion gives a remarkably clear sound over the chest. Auscultation detects the *bruit de souffle* in the carotids. Has a bad cough, short and dry; complexion sallow to excess; cheek bones projecting; cheeks sunken; tongue thickly coated, white, with red tip, and edges; bowels irregular; epigastric tenderness; limbs attenuated. His anaemic condition led me, at first sight, to suppose the man a far advanced case of phthisis, and I was never as much disappointed as when the chest proved resonant, and remarkably so, throughout. But his extreme prostration, and the apparent total destruction of the function of nutrition, seemed to offer as little promise, as if his case had been, as at first, supposed. To satisfy him, I prescribed the Strychnia, with the hope that it might benefit him. I ordered him one-twelfth of a grain to be taken thrice, daily.

Dec. 1st. Is much better; cough has nearly ceased; has been clear from chill from the fourth day, until yesterday, when he got his feet a little wet and had a chill; appetite something better; the sense of taste which was destroyed, is, in a measure, returning. I directed him to increase the dose of Strychnia to one-eighth of a grain for each dose, and to continue as before.

15th. Continues growing better. I directed him to continue the Strychnia until two grains more are taken.

Jan. 1848. He has recovered entirely.

Case 6. Mrs. O. B., æt. 34, wife of the man whose case is given above, had the fever at the same time as her husband. She applied to me on the same day. She has now rigors and fevers every second day; tongue coated white; bowels lax, with occasional severe diarrhœa; is much emaciated; appetite very poor; pulse irregular and quick; intermitting pain over the left eye. I directed for her, Strychnia, grain, one-eighth to be taken three times each day.

Nov. 20th. Rigors arrested, but the Strychnia was prescribed as before, to guard against a relapse. It was taken, in all, eight days.

Case 7. Mrs. McM., æt. 48; a case of complication with bronchitis, sent for me on December twenty-second. Her health has never been very good. She has been subject all her life to occasional attacks of bronchitis; sometimes, in the spring, particularly, to severe attacks. She has been confined to her bed now for two months. She has a very severe cough with copious expectoration of frothy mucous; respiration laborious; tongue, deeply coated, red edged; pain in epigastrium and left hypocondrium; bowels rather costive. Percussion gives a very clear sound over the chest. Auscultation, the respiration is distinct over the whole chest; but the bronchia are loaded with mucous. She has had a chill for nearly three months, which commences at about two o'clock, P. M., and continues for three or four hours, followed by fever and copious diaphoresis; sleeps scarcely any at all; has no appetite whatever, and is obliged to force herself to take any food; great thirst; is very much reduced in flesh and is scarcely able to move in bed; when she attempts to turn, the effort brings on palpitations that are very distressing. Has severe pain constantly over both eye-brows. I directed her two grains of quinine to be given three times each day.

23. Rested better last night; pain in the head better; chill lighter, to-day. I directed the quinine to be continued.

25th, A. M. Yesterday she felt quite well, comparatively; feels stronger; appetite no better; bowels still costive. I directed castor oil.

25th, P. M. Is much worse; pain in the head very severe; has had cold chills all day and cold perspirations; cough much more troublesome; expectoration more labored; the oil operated very well. I directed her one-twelfth grain of Strychnia, three times each day.

26th. Tells better; slept well last night; has no cold perspirations to-day; continue Strychnia.

28th. Better; continue Strychnia.

29th. Rested well last night; no chill yesterday at all; eat with some relish, a little gruel, yesterday. I directed the Strychina to be increased to one-eighth of grain for each dose.

31st. Has been much better yesterday and to-day ; no chills ; yet the cold perspirations continued on the lower part of the body. The bronchial secretion has been gradually diminishing with the returning strength. Continue Strychnia.

January 11th, 1848. Has been able to set up during the past week ; her appearance much improved ; cough not so troublesome ; expectoration still diminishing gradually ; chills gone entirely, but the cold perspiration still a little troublesome. She eat a hard boiled egg yesterday, which caused her much annoyance. Directed her a dose of castor oil, and after its operation, nitrate of potash, four grains, to be taken four times each day until the perspiration ceases.

13th. Perspirations have ceased ; Strychnia resumed until two grains are taken.

17th. Still continues to grow better ; appetite good ; scarcely any cough or expectoration.

Observations.—The cases might be continued ; but as the others that I have entered in my case book, present the same general features with those here given, their notice is unnecessary. In the foregoing, I have been careful to observe the condition of the lungs : for in the four cases in which I tried the Strychnia, without any evident signs of benefit, there existed phthisis. Of these cases it is unnecessary to say more, than that the chills occurred every evening, and were not in the slighest perceptible degree, controlled by the remedy ; although, in one case, it was continued for three weeks, and I am, therefore, inclined to believe, that the *integrity of the lungs is necessary to the beneficial action of the Strychnia in the restoration of the function of nutrition and secretion.* Of the physiological action of the remedy I say nothing. I merely give the cases as they occurred, with the treatment and the result. Facts at this stage of the inquiry as to Strychnia, are what are wanted. That Strychnia posesses the power of arresting intermittent fever, is certain ; and that its effects are, though slower, more permanent than the effects of quinine, is equally certain. Besides, it possesses this great advantage over quinine; that while the quinine, in cases of deranged digestive function, as of case 5, would rather increase the difficulty, and surely not benefit, the Strychnia produced marked and permanent amelioration. I may be allowed to remark that, to me, the results here communicated were peculiarly satisfactory, for these were the very kind of cases that I and my medical brethren, in the district before mentioned, found most difficulty in managing.

I believe that Strychnia will take its rank as an adjuvant to quinine, in the treatment of fevers; and, that, while the latter will be used, as now, to break up the paroxysms, the former will then take its place to perpetuate the cure, restoring the perverted nutrition or deranged secretion, the cause or consequence, as the case may be, of disease. I am satisfied that the Strychnia is, by no means, as dangerous a remedy as is generally supposed. Case three, took six and two-third grains, in twenty-three days. Case five, took eleven and one-eighth grains, in forty-one days; and, case seven, took eight and a fourth grains in twenty-seven days. In all the cases the effects of the remedy were closely watched in order to observe the least symptom that might contra-indicate its use. It produced slight tetanic spasms in case one, and also slight vomiting when first given. In two cases the patients complained of vertigo. These were the only effects observed and they were not considered sufficient to require the Strychnia to be discontinued. It was always given in powder mixed with starch.

*Quere*? As both the cases, three and five, in which anæmia was present, in so marked a degree, were salivated profusely, during their treatment, might not this condition of anæmia have been caused or, at least, aggravated by the free and indiscriminate use of mercury? We know that mercury will combine with the pepsin, thereby impairing, for a time, digestion; and if a fresh portion of mercury be still added to neutralize each new portion of pepsin eliminated, we can easily see that it would be very easy to perpetuate its destructive effects upon digestion, and more particularly so, since the pathological tendency of the disease itself is so strong in that direction. If such be the case, might not many physicians do better than to indiscriminately administer mercury in these fevers!

